# Supplementary material for: Lipopeptides as rhizosphere public goods for microbial cooperation
Source: Microbiol Spectr. 2023 Dec 4;12(1):e03106-23. doi: 10.1128/spectrum.03106-23 (PMC10783051; doi:10.1128/spectrum.03106-23)
Supplement: Supplemental material — Fig. S1 to S13 and Tables S1 to S4. [file spectrum.03106-23-s0001.docx]

# Supplementary figures and tables

**
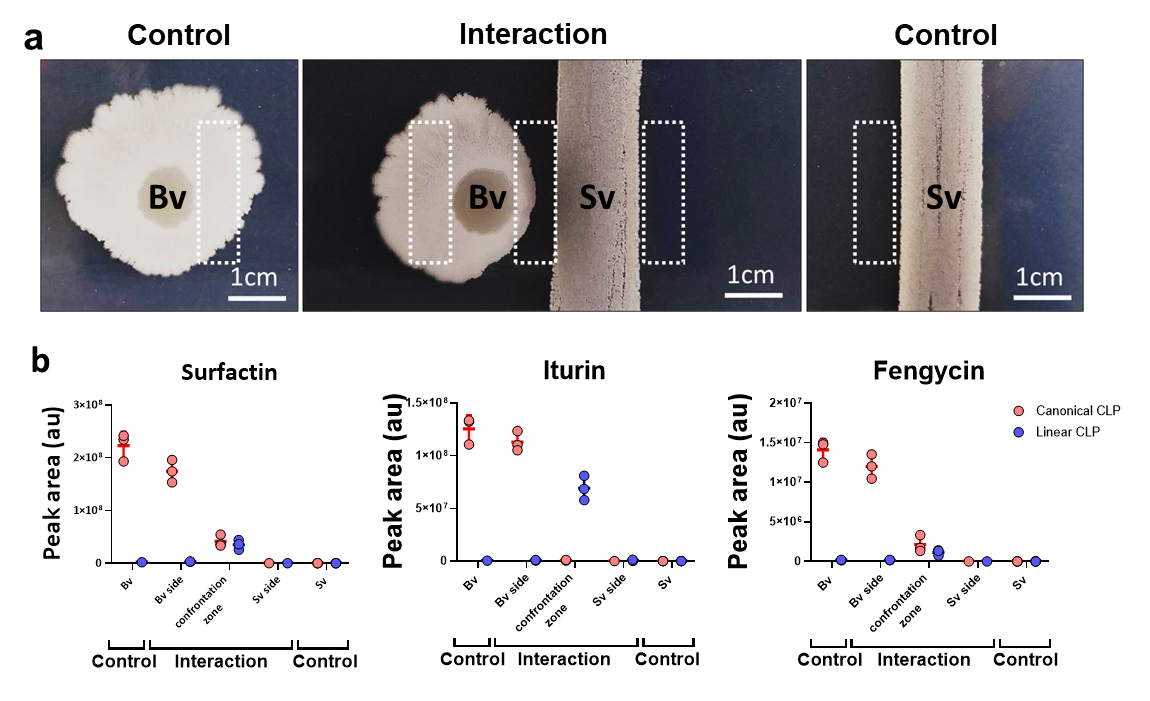
**

**Supp. fig. 1. Degradation of *Bacillus* lipopeptides surfactin, iturin and fengycin in interaction between *Bacillus velezensis* GA1 and *Streptomyces venezuelae* ATCC 10712. a.** Pictures of, from left to right, *Bacillus velezensis* GA1 alone (Bv), the interaction between *B. velezensis* (Bv, left side) and *S. venezuelae* (Sv, right side) and *S. venezuelae alone* (Sv). Dashed squares represent the sampling areas for metabolites extraction. Pictures are representative of 3 biological replicates. **b.** Mean peak areas of canonical (blue) and linear (red) CLPs of *Bacillus* (surfactin, iturin and fengycin) extracted from agar in the control *Bacillus velezensis* GA1 (Bv), the control *S. venezuelae* (Sv) and in coculture of *B. velezensis* and *S. venezuelae*: on the side of *B. velezensis* (Bv side), in the interaction zone in-between Sv and Bv (confrontation zone) and on the *S. venezuelae* side (Sv side) as represented by the dashed squares in the pictures panel a. n=3 biological replicates. error bars indicate ± standard deviation. Peak areas were measured from UPLC-ESI-MS EIC of the monoisotopic ions [m+H]^+^.


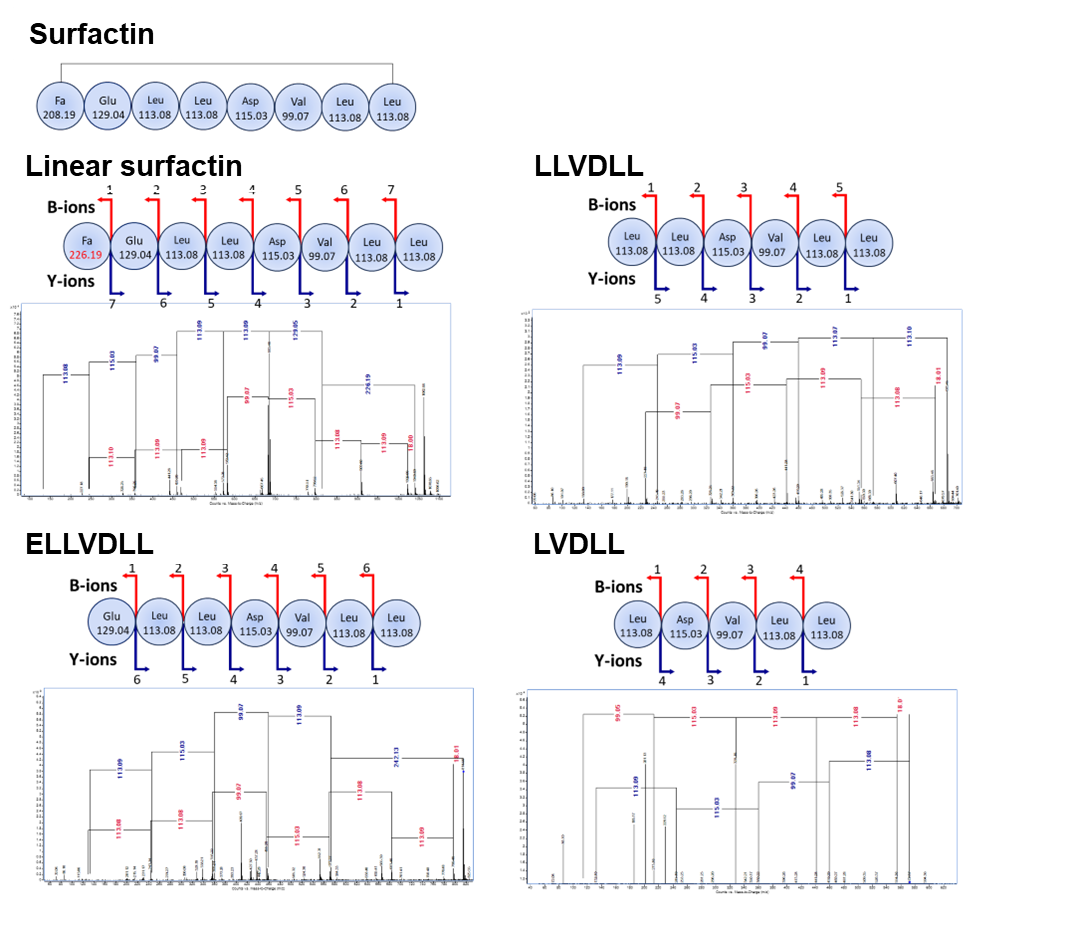


**Supp fig. 2. UPLC-ESI-qTOF MS/MS spectra of surfactin degradation products generated by Sv.** Clippers represent B- and Y- ions sequences (in blue and red respectively). MS/MS spectra are merged spectra acquired at CID energy 20 and 40V.

**
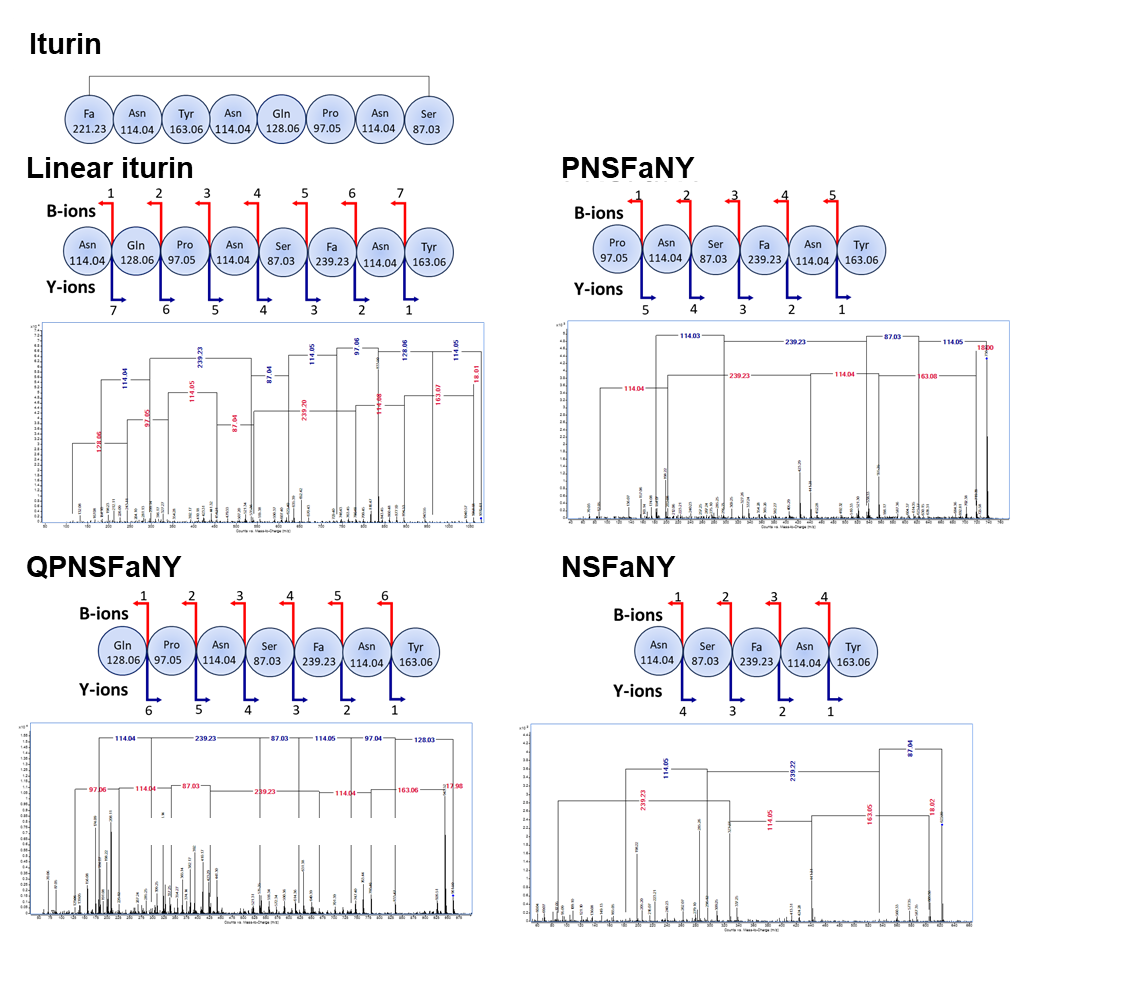
**

**Supp. fig. 3. UPLC-ESI-qTOF MS/MS spectra of iturin degradation products generated by Sv.** Clippers represent B- and Y- ions sequences (in blue and red respectively). MS/MS CID energy was 50V**.**

**
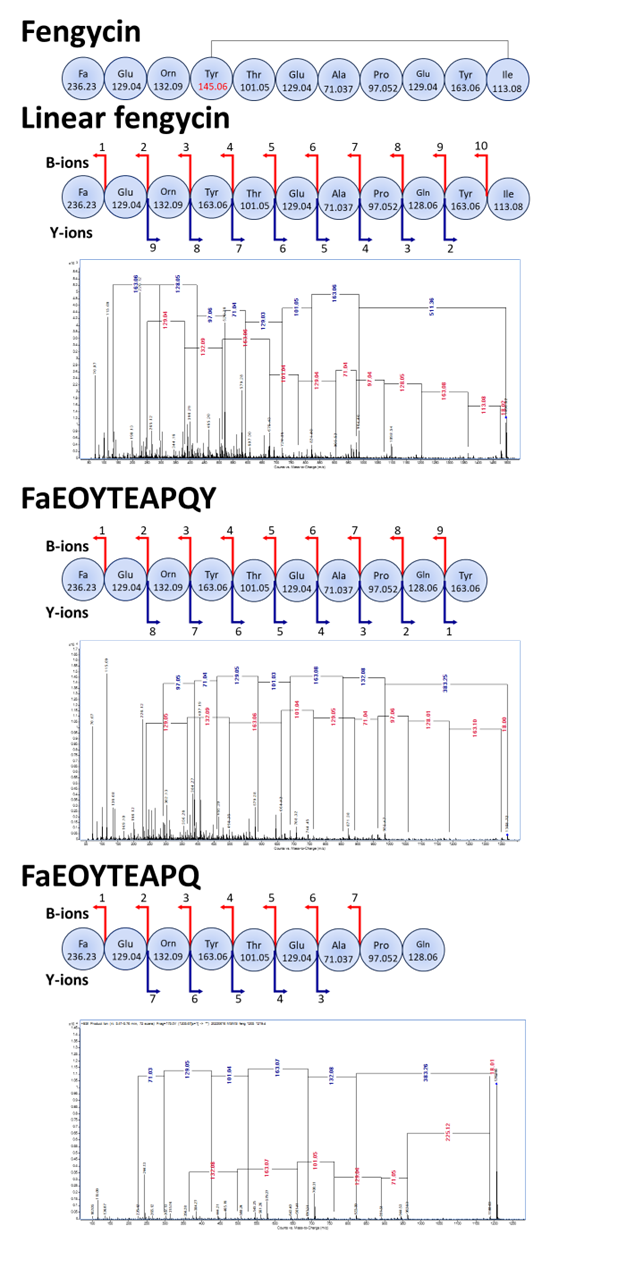
**

**Supp. fig. 4. UPLC-ESI-qTOF MS/MS spectra of fengycin degradation products generated by Sv.** Clippers represent B- and Y- ions sequences (in blue and red respectively). MS/MS CID energy was 60V**.**

**Supp. fig. 5. Degradation kinetics of surfactin, iturin and fengycin in presence of Sv supernatant.** Canonical CLPs and degradation products contents were measured by UPLC-ESI MS and are expressed as peak area. error bars represent the standard deviation, n=3. Degradation kinetics were performed on 40µM pure surfactin, iturin and fengycin incubated at 30°C for 48h with 10% (v/v) filter-sterilized (0.22µM filters) *S. venezuelae* supernatant grown on ISP2 (adequate for enzyme production).


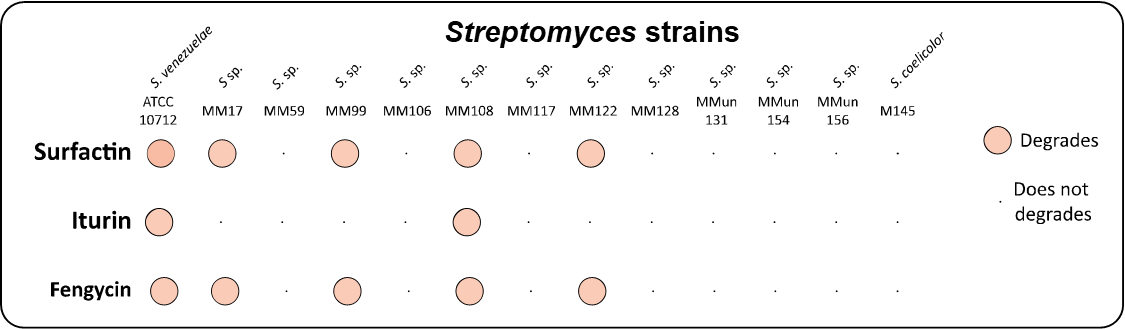


**Supp. fig 6. Ability of *Streptomyces* strains to degrade *Bacillus* CLPs**. Bv supernatant was incubated with 4% (v/v) of *Streptomyces* supernatant of each strain grown on ISP2. Degradation products contents were assessed by UPLC-ESI MS. Each experiments were performed on triplicate.


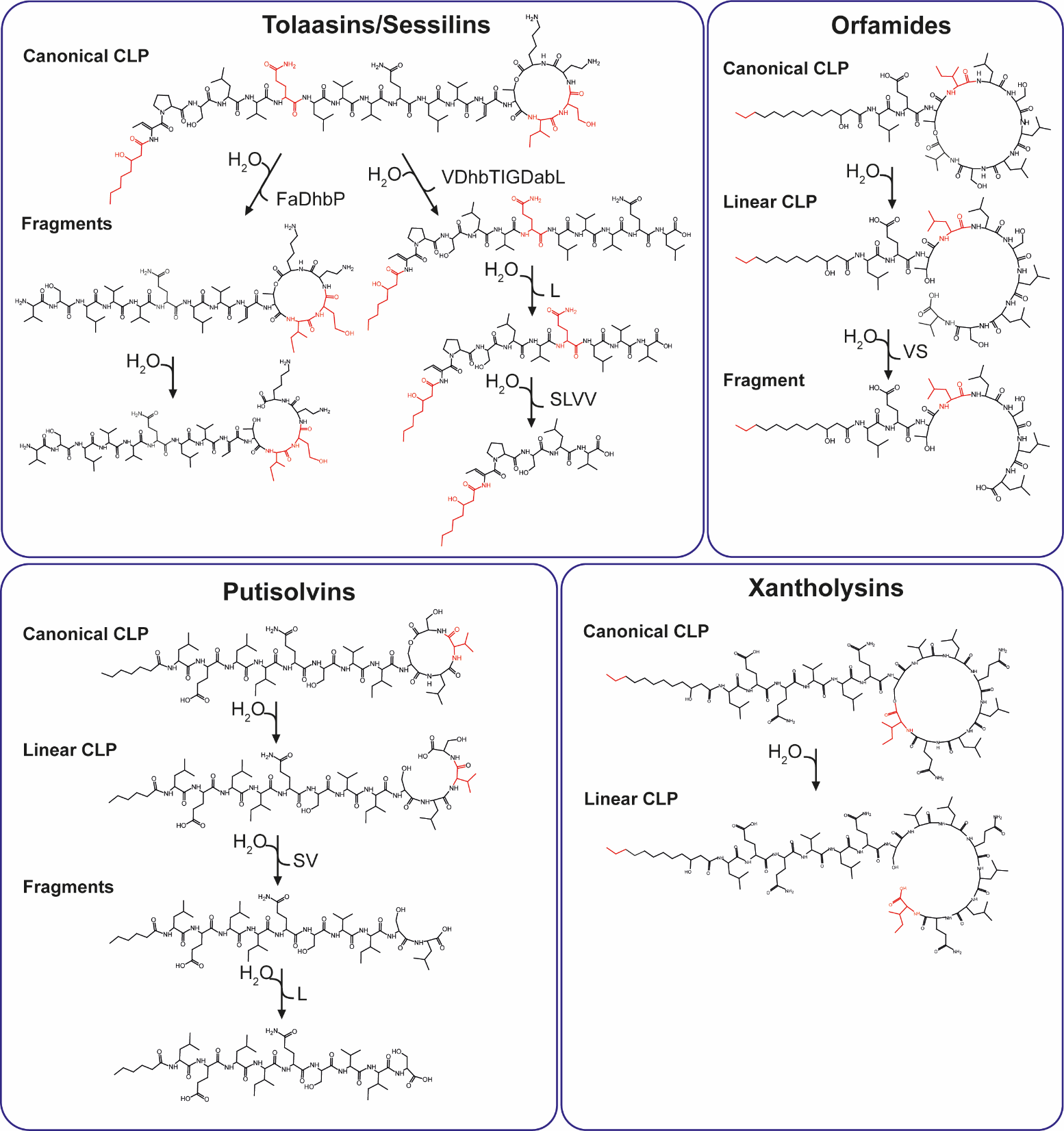


**Supp. fig. 7.** **Proposed degradation mechanisms of *Pseudomonas* spp. CLPs tolaasins, sessilins, orfamides, putisolvins and xantholysins by *S. venezuelae.*** The mechanisms are inferred from the fragments detected and identified of each CLPs. To generate and identify degradation products, CFS of *Pseudomonas* containing the CLPs were incubated for 24 h at 30°C supplemented with 4% (v/v) of Sv CFS. The samples were then analyzed by UPLC-ESI-MS and structures were determined by UPLC-ESI-MS/MS. MS/MS spectra of the fragments are available in supp. Fig. 7-9.


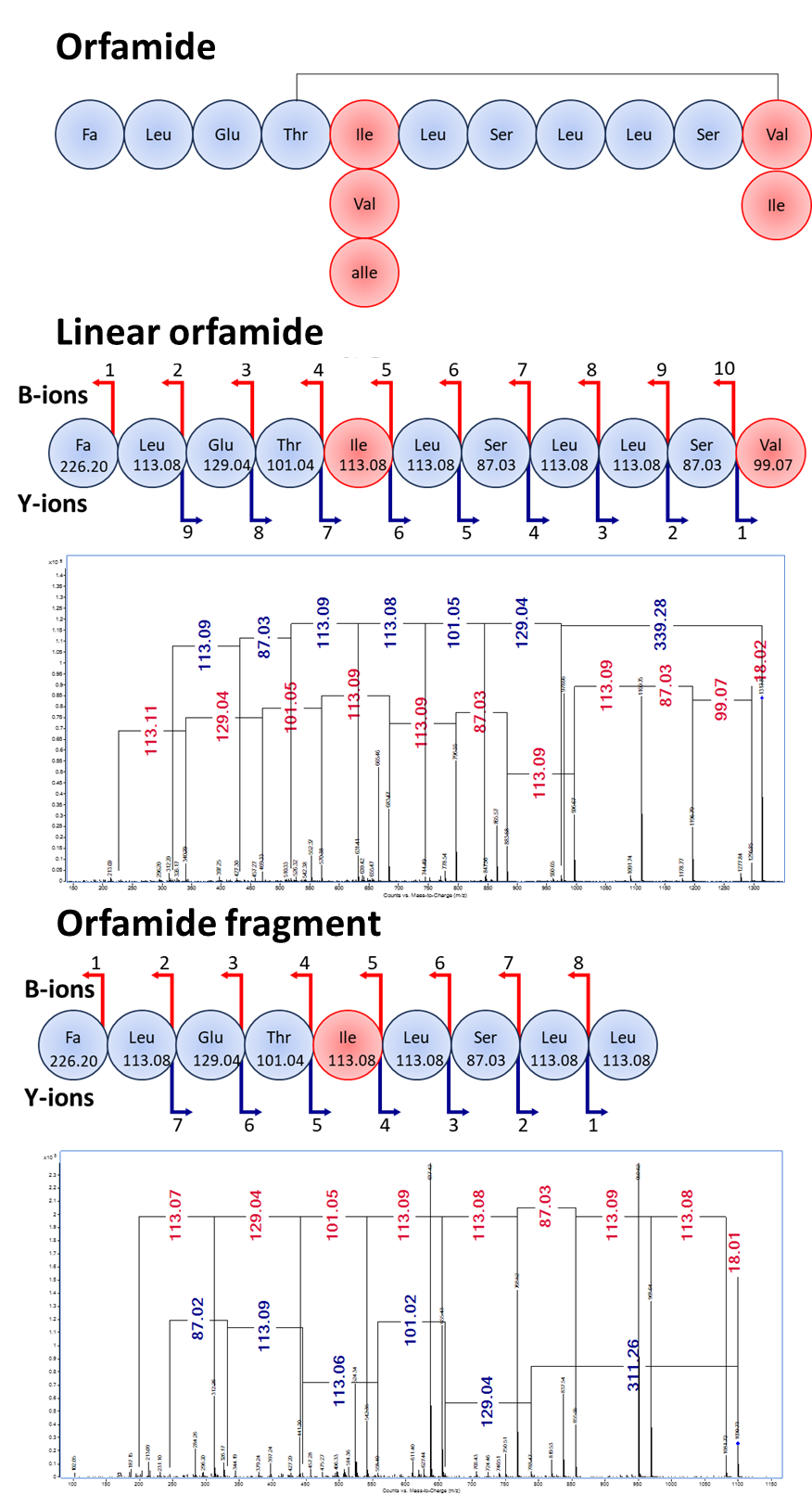


**Supp. fig. 8. UPLC-ESI-qTOF MS/MS spectra of orfamide degradation products generated by Sv.** Clippers represent B- and Y- ions sequences (in blue and red respectively). MS/MS CID energy was 20 and 40V**.** Red balls correspond to the positions of amino acid substitutions in the orfamide variants.

**
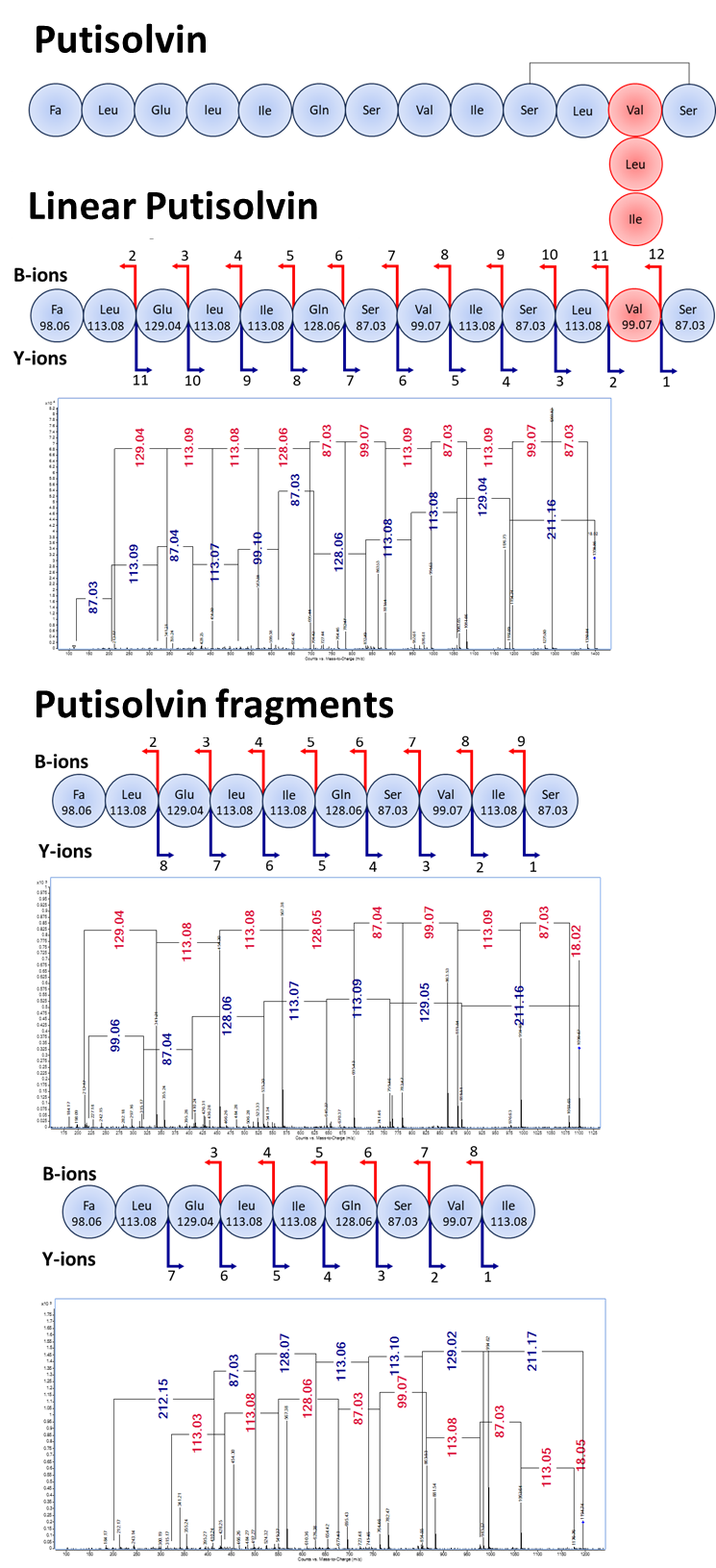
**

**Supp. fig. 9. UPLC-ESI-qTOF MS/MS spectra of putisolvin degradation products generated by Sv.** Clippers represent B- and Y- ions sequences (in blue and red respectively). MS/MS CID energy was 20 and 40V **.** Red balls correspond to the positions of amino acid substitutions in the putisolvin variants

**
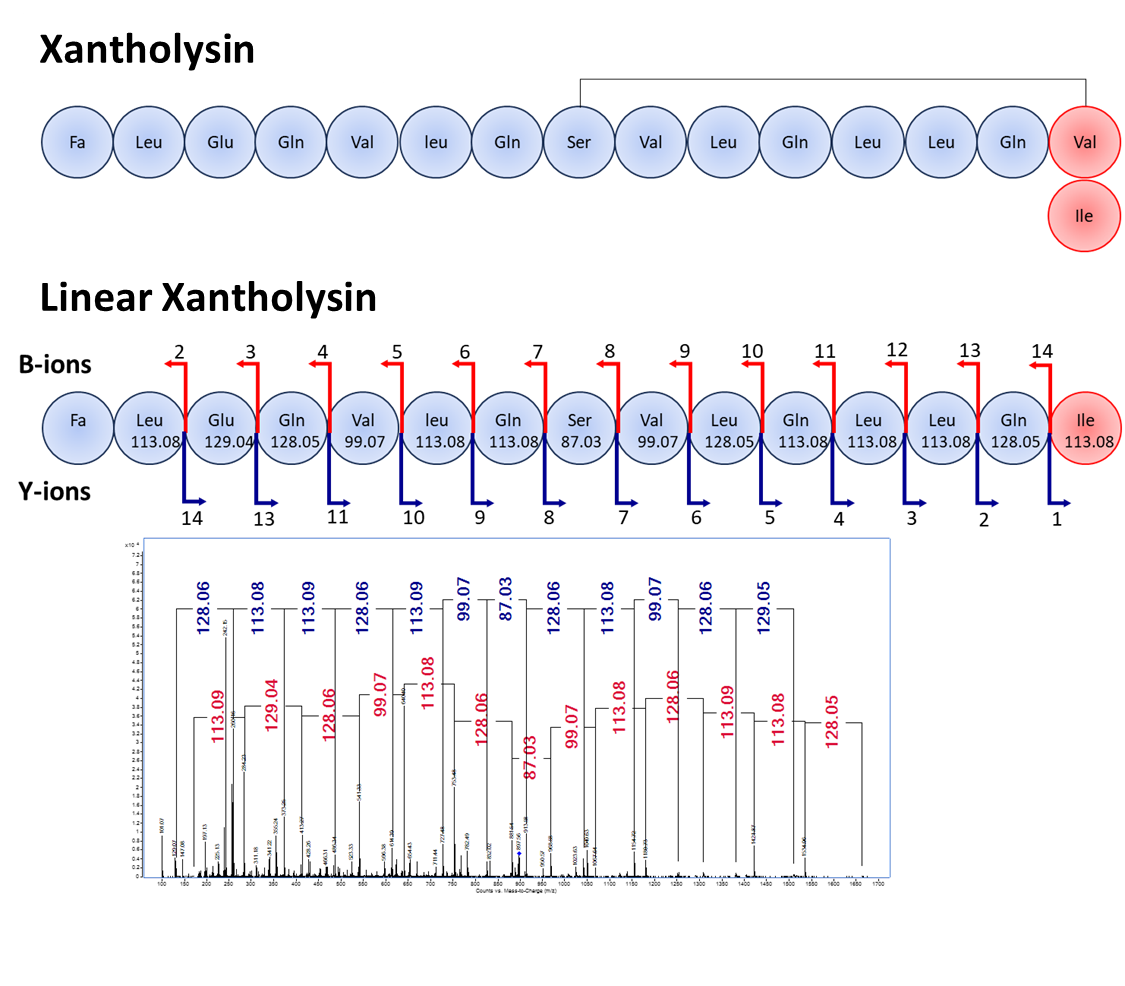
**

**Supp. fig. 10. UPLC-ESI-qTOF MS/MS spectra Xantholysin degradation products generated by Sv.** Clippers represent B- and Y- ions sequences (in blue and red respectively). MS/MS CID energy was 20 and 40V **.** Red balls correspond to the positions of amino acid substitutions in the Xantholysin variants.


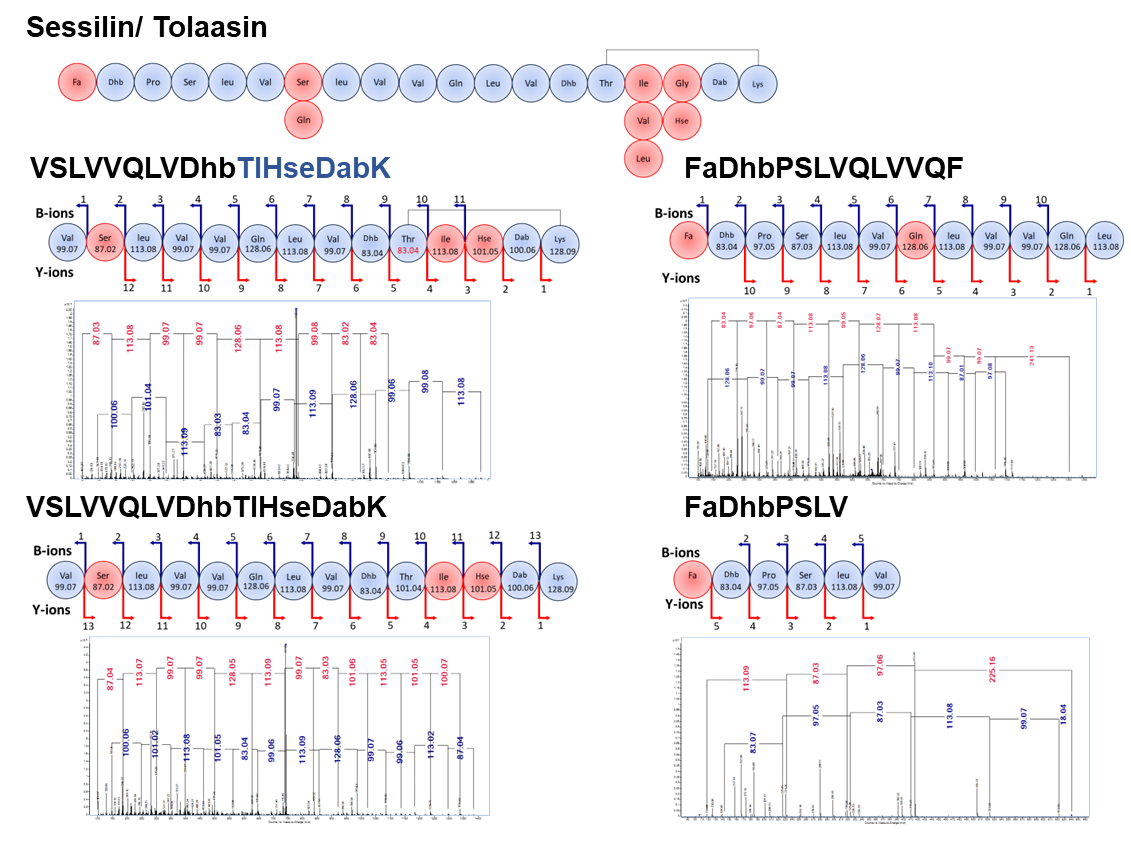


**Supp. fig. 11. UPLC-ESI-qTOF MS/MS spectra of sessilin/tolaasin degradation products generated by Sv.** Clippers represent B- and Y- ions sequences (in blue and red respectively). MS/MS CID energy was 20 and 40V **.** Red balls correspond to the positions of amino acid substitutions in the sessilin/tolaasin variants. Letters in blue in the peptide chain (VSLVVQLVDhbTIHseDabK) correspond to the amino acids in the peptide cycle.


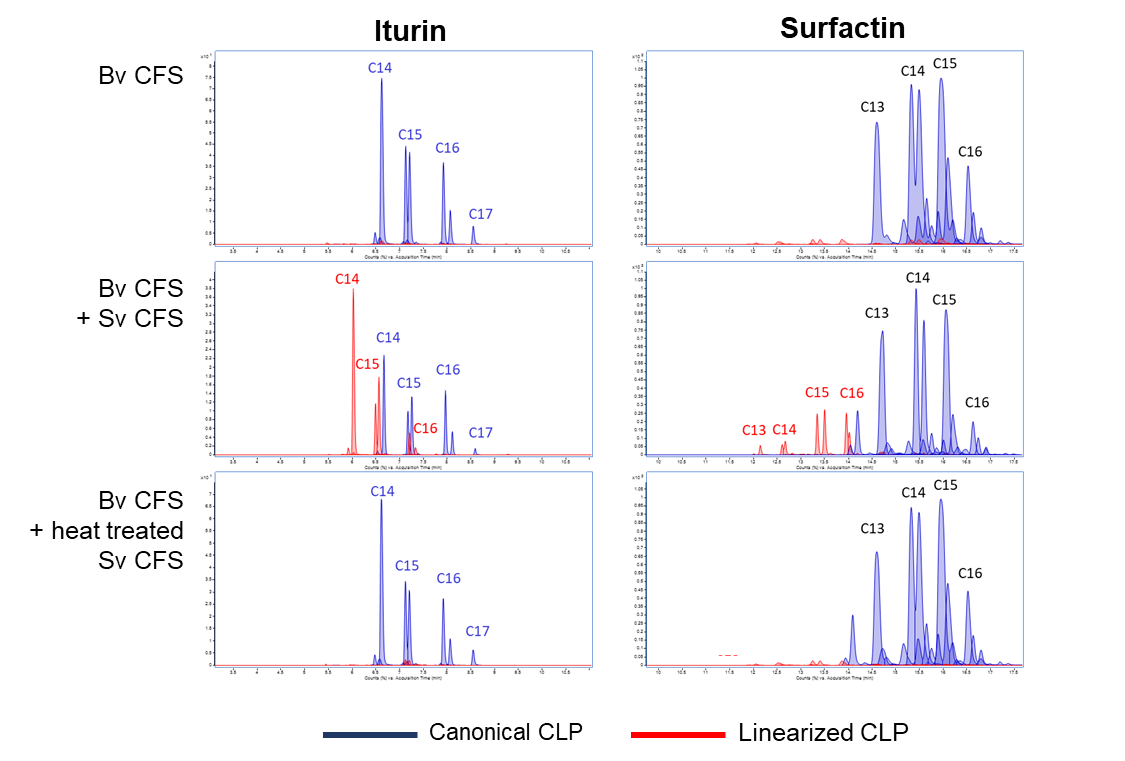


**Supp. fig. 12. The CLP-degradation activity of S. venezuelae is heat sensitive.** LC-MS EIC of canonical and linear iturin (left) and canonical and linear surfactin (right) in *B. velezensis* GA1 supernatant (top), *B. velezensis* GA1 supplemented with *S. venezuelae* ATCC10712 (middle) and *B. velezensis* GA1 supernatant supplemented with *S. venezuelae* ATCC10712 heat treated supernatant (10’ at 98°C, bottom). Notation “Cn” corresponds to the fatty acid chain length of the variants of surfactin and iturin associated to each peak. Y-axes of the chromatograms of the 3 conditions are linked for iturin and surfactin.

**
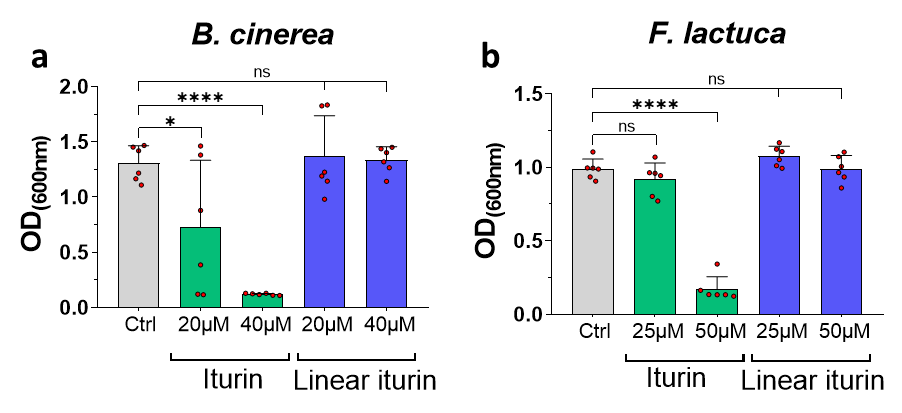
**

**Supp. fig. 13. Degraded iturin losses its inhibitory activity against fungal phytopathogens.** Optical density of *B. cinerea* and *F. lactuca* liquid cultures supplemented with canonical or degraded iturin*.* Graphs show the mean optical density (OD_600nm_) and ±SD calculated for 6 biological replicates (n=6). OD_600nm_ was measured on 96 wells microplates inoculated with 10^6^ spores/ml and grown for 96 and 48h for *Botrytis* and *Fusarium* respectively. Ctrl corresponds to fungal culture without (linear)iturin supplementation. Statistical comparison between control and supplemented with CLPs was performed based on T-test (ns: not significant, *: p<0.05, **** p<0.0001).

**Supp. table 1. Stains used in this study**

**Supp. table 2.** MZmine parameters used for FBMN

**Supp. table 3. Secreted proteins found only in active Sv CFS with function possibly related to CLP catabolism**. The content in protein in conducive medium (ISP6, i.e. medium on which Sv CFS degrades Bv CLPs) was compared to non-conducive medium (ISP5).

**Supp. table 4. Features identified in Fig. 1 a,b,c.**
